# Complete mitochondrial genome of the Ruff*, Calidris pugnax* (Aves, Scolopacidae)

**DOI:** 10.1080/23802359.2020.1731356

**Published:** 2020-02-28

**Authors:** Tai-Yu Chen, Fang Zhang, Guo-Hai Wang, Man-Yu Zhang, Tao Liang, Chang-Hu Lu

**Affiliations:** College of Biology and the Environment, Nanjing Forestry University, Nanjing, Jiangsu, China

**Keywords:** *Calidris pugnax*, mitochondrial genome, Ruff, Scolopacidae

## Abstract

This paper reports the mitochondrial genome of *Calidris pugnax*, which is a closed circular molecule of 16,902 bp. The overall base composition of this species is 25.2% T, 30.6% C, 32.0% A, and 12.2% G, with an A + T content of 55.2%. The structure of the mitogenome is a typical vertebrate mitochondrial gene arrangement. Phylogenetic analysis of complete mitochondrial genome concatenated sequences from 13 species from 6 genera was conducted using the maximum-likelihood (ML) model. The topology demonstrated that the mitogenome of this species was genetically closest to that of *Calidris tenuirostris*. *Calidris pugnax* mitogenome can contribute to our understanding of the phylogeny and evolution of this species.

Ruff (*Calidris pugnax*), which belongs to the genus *Calidris* within the family Scolopacidae (Aves, Charadriiformes), mainly breeds in Northern Eurasia and migrates to Africa, southeast Asia, and Australia during winter. Ruff is a single species with no subspecies, and was placed in monotypic genus *Philomachus* before, but a recent study indicates that the latter is embedded within *Calidris* (Gibson and Baker 2012). Ruff is special among Scolopacidae bird species, and the morphology of males are diverse during the breeding period. In this study, we determined the complete mitochondrial genome (mt genome) of *C. pugnax* for the first time. The samples were collected at the Bird Circle Station of the Guiyang Longdongbao International Airport (26°32.983′N, 106°48.585′E), Guizhou province, China in December 2019. After sampling, the specimens (NJFU-2020-LSY) were stored in the Animal Specimens Museum of Nanjing Forestry University. Genome DNA was isolated from paw tissue using a kit and 12 primers were designed for fragment amplification.

The complete mitochondrial genome of *C. pugnax* is a closed circular molecule composed of 16,902 bp (GenBank accession no. MN956840), containing the typical set of 13 protein-coding genes (PCGs), 22 transfer RNA genes, 2 ribosomal RNA genes (*rrnS* and *rrnL*), and one control region (D-loop). The nucleotide composition is 25.2% T, 30.6% C, 32.0% A, and 12.2% G, with an A + T content of 55.2%. The structure of the mitogenome is a typical vertebrate mitochondrial gene arrangement (Noack et al. 1996; Liu, Yang et al. 2019; Sun et al. 2019).

In this study, phylogenetic analysis of the *C. pugnax* and 13 other Scolopacidae bird species was carried out based on the concatenated nucleotide sequences of the complete mitochondrial genome using the maximum-likelihood (ML) model in MEGA 7.0 (Kumar et al. 2016) with 1000 bootstrap replicates. In this phylogenetic tree, *C. pugnax* is clustered with *C. tenuirostri*, which indicates that they have a much closer relationship (Figure 1), The phylogenetic tree reveals that the data of our new determined mitogenome explain some related evolution issues. It appears that *C. pugnax* is more closely related to the birds of the genus *Calidris*, the phylogenetic analysis result was consistent with the previous research (Gibson and Baker 2012). *Calidris pugnax* mitogenome can contribute to our understanding of the phylogeny and evolution of this species. (Boore 1999; Liu, Xu et al. 2019; Sun et al. 2020).

**Figure 1.**
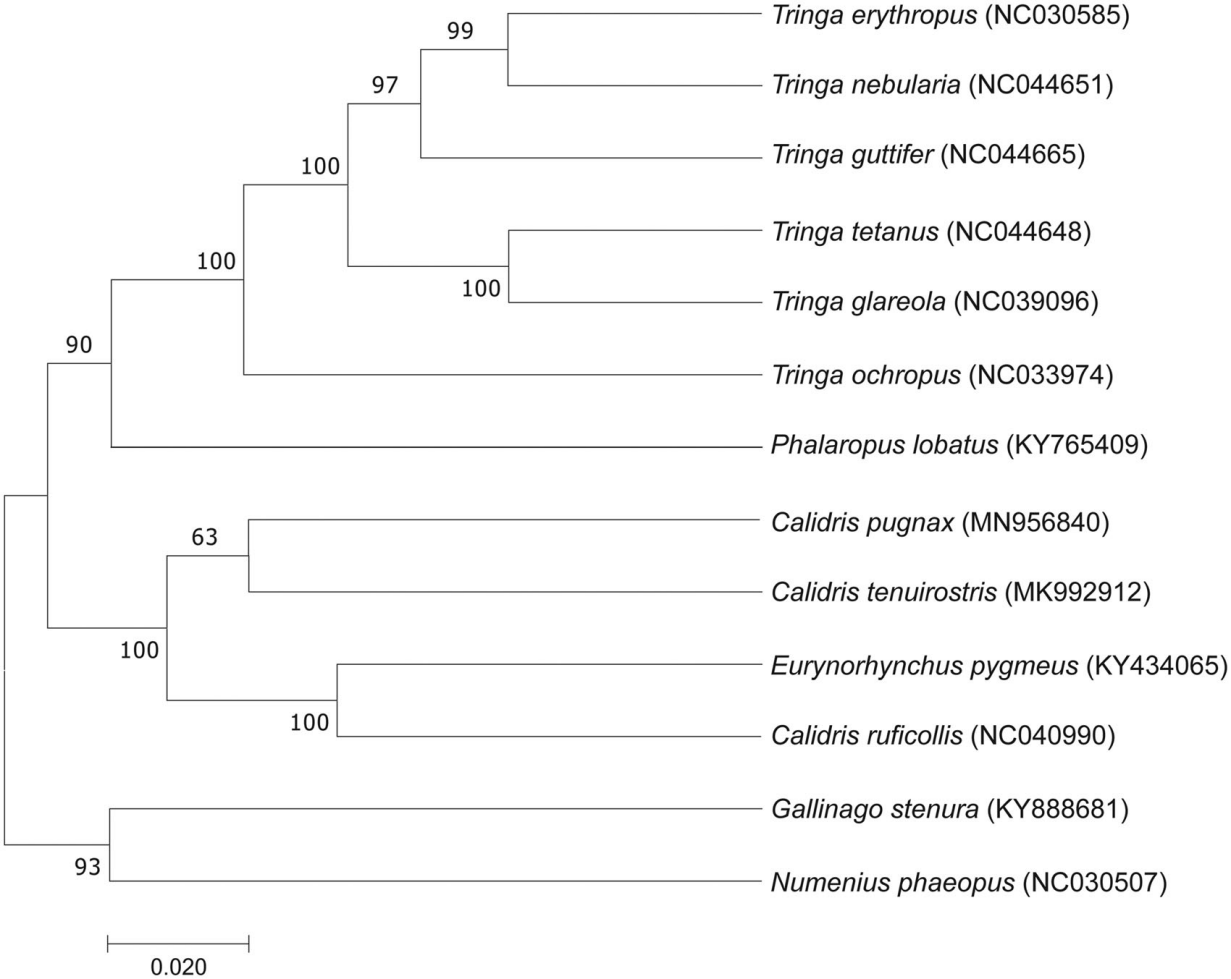
Maximum-likelihood (ML) tree of based on the mitochondrial genome sequences of *C. pugnax* and other 12 species from Scolopacidae birds using MEGA 7.0.
